# Improvement of PTSD-like behavior by the forgetting effect of hippocampal neurogenesis enhancer memantine in a social defeat stress paradigm

**DOI:** 10.1186/s13041-019-0488-6

**Published:** 2019-08-02

**Authors:** Rie Ishikawa, Chiaki Uchida, Shiho Kitaoka, Tomoyuki Furuyashiki, Satoshi Kida

**Affiliations:** 1 Department of Bioscience, Faculty of Applied Bioscience, Tokyo University of Agriculture, 1-1-1 Sakuragaoka, Setagaya-ku, Tokyo, 156-8502 Japan; 2 Division of Pharmacology, Kobe University Graduate School of Medicine, 7-5-1 Kusunoki-cho, Chuo-ku, Kobe, Hyogo 650-0017 Japan; 3 Graduate School of Agriculture and Life Sciences, The University of Tokyo, 1-1-1 Yayoi, Bunkyo-ku, Tokyo, 113-8657 Japan

**Keywords:** PTSD, Adult hippocampal neurogenesis, Social defeat, Forgetting, Anxiety-like behavior

## Abstract

Post-traumatic stress disorder (PTSD) is a psychiatric disorder associated with memories of traumatic experiences. Recent studies have shown that the forgetting of contextual fear memory is promoted via increased adult hippocampal neurogenesis induced by neurogenesis enhancers, such as memantine (MEM) and exercise, raising the possibility that neurogenesis enhancers improve PTSD by facilitating the forgetting of traumatic memory. On the other hand, repeated exposure to social defeat (SD) stress by aggressor mice induces social avoidance behavior to the aggressor and chronic anxiety-like behavior. In this study, we assumed this SD stress paradigm as a PTSD-like model and examined the effects of treatment with neurogenesis enhancer MEM on SD stress-induced PTSD-like behavior. Male C57BL/6 mice received SD stress for 10 consecutive days and were assessed for social avoidance memory to the aggressor (memory of aggressor mice) and anxiety-like behavior using social interaction and elevated zero maze tasks. Consistent with previous studies, SD mice formed social avoidance memory and exhibited increased anxiety-like behavior. Importantly, subsequent MEM treatment (once a week for 4 weeks) significantly reduced social avoidance behavior, suggesting that MEM-treated SD mice showed forgetting of social avoidance memory. Interestingly, MEM-treated SD mice showed comparable anxiety-like behavior with control mice that were not exposed to SD stress. Moreover, MEM-treated SD mice showed no reinstatement of social avoidance memory following single re-exposure to the aggressor. Our findings suggest that neurogenesis enhancer not only enhanced the forgetting of traumatic memory but also improved PTSD (anxiety)-like behavior.

## Maintext

Post-traumatic stress disorder (PTSD) is a mental disorder associated with traumatic memory such as fear memory. Importantly, fear memory processes, including memory reconsolidation and extinction, have been thought to be therapeutic targets for PTSD [1–4]. Previous studies have shown that adult hippocampal neurogenesis enhancers, such as exercise and memantine (MEM), an uncompetitive antagonist of the *N*-methyl-_D_-aspartate glutamate receptor, promote hippocampus-dependent fear memory forgetting in mice [5, 6], raising the possibility that fear memory forgetting could be a novel therapeutic target for PTSD [4].

In the social defeat (SD) stress paradigm in rodents, a mouse is attacked repeatedly by aggressor mice [7]. This SD paradigm may be similar with traumatic experiences. Interestingly, repeated exposure to SD stress by aggressor mice induces not only social avoidance behavior to the aggressor (memory of the aggressor mice) but also chronic anxiety-like behavior [8–10]. Therefore, we assumed that social avoidance and chronic anxiety-like behavior reflect PTSD-like states consisting of impaired emotional behavior associated with traumatic social memory. In this study, we used this SD stress paradigm as a PTSD-like model and examined the effects of treatment with neurogenesis enhancer MEM on SD stress-induced PTSD-like behavior.

**Fig. 1:**
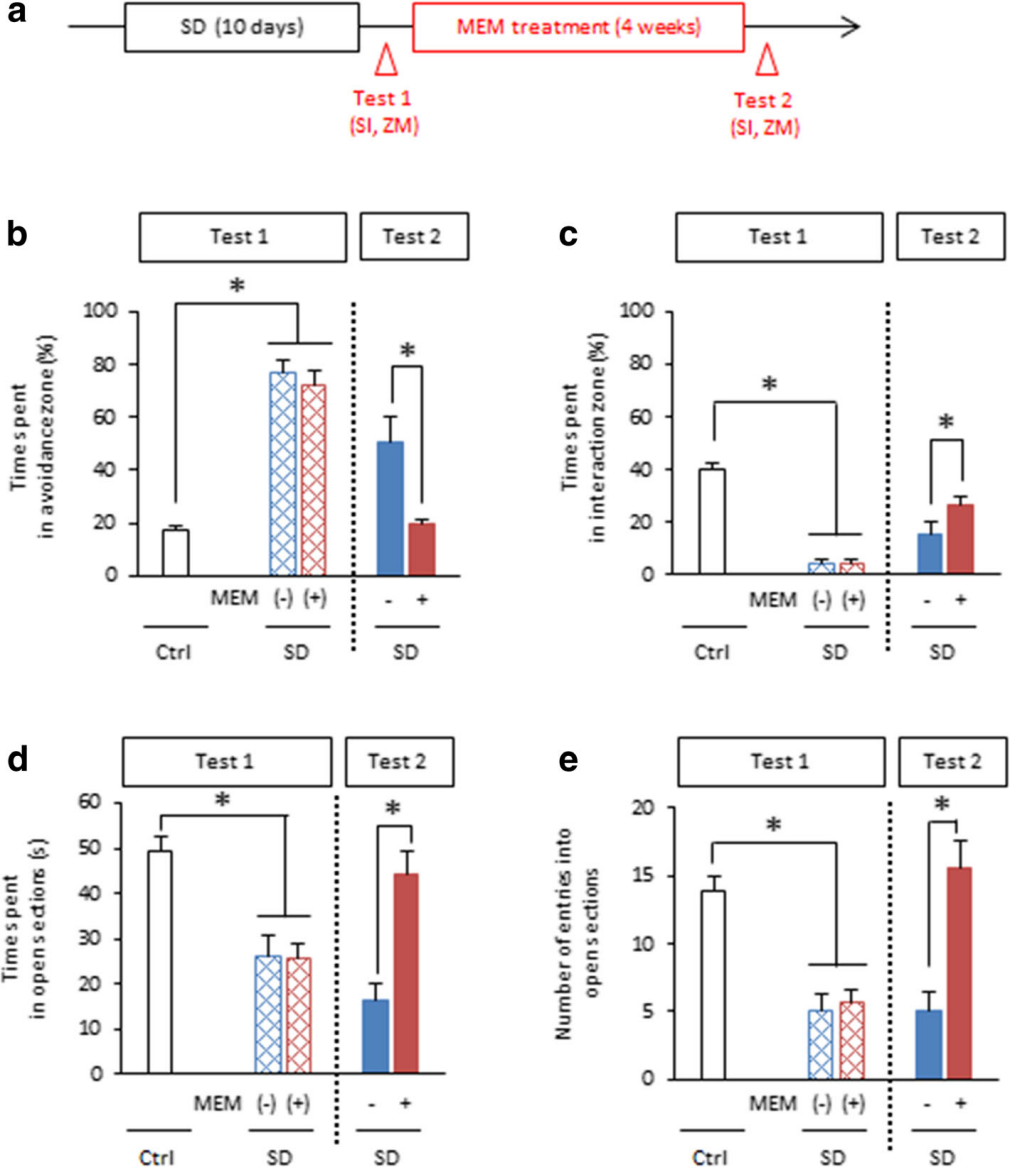
MEM enhances forgetting of social avoidance memory and improves anxiety-like behavior induced by SD stress. **a** Experimental procedure. SI, social interaction task; ZM, elevated zero maze task. **b** and **c** Social interaction task in Test 1 and 2 (Ctrl, *n* = 35; VEH-SD, *n* = 14; MEM-SD, *n* = 15). Time spent in the avoidance zone (**b**) and interaction zone (**c**). **d** and **e** Elevated zero maze task (Ctrl, n = 35; VEH-SD, n = 14; MEM-SD, n = 15). Time spent in the open sections (**d**) and number of entries into the open sections (**e**). *p < 0.05. Error bars indicate SEM

In this SD treatment, male C57BL/6 mice were exposed to an aggressor ICR mouse for 10 min per day for 10 consecutive days (SD mice). At 24 h after the last exposure to SD stress, we performed social interaction and elevated zero maze tasks to assess social avoidance and anxiety-like behavior, respectively, in SD mice (Test 1; Fig. 1a). Control mice were not exposed to SD treatment but tested as SD mice. Subsequently, SD and control mice received systemic injections of MEM (50 mg/kg body weight) or vehicle (VEH) once a week for 4 weeks as our previous experiments [6] and were assessed again for social avoidance and anxiety-like behavior at a week after the last MEM injection (Test 2).

In the social interaction task, mice were assessed for time spent in interaction (close) and avoidance (distant) zones from an aggressor, respectively, in an open field in which an encaged aggressor mouse was placed in the center of interaction zone. SD mice exhibited significantly more and less time spent in the avoidance and interaction zones, respectively, than no SD (control) mice during Test 1 (one-way analysis of variance [ANOVA]: avoidance zone, *F*_(4, 92)_ = 60.144, *p* < 0.05; interaction zone, *F*_(4, 92)_ = 43.07, *p* < 0.05; Fig. 1b, c). These observations indicated that SD mice showed social avoidance behavior, suggesting that they formed social avoidance memory.

We then performed an elevated zero maze task to examine anxiety-like behavior. In this task, the time spent in the open sections and the number of entries into the open sections are thought to reflect their state of anxiety, since mice generally avoid high open spaces. SD mice exhibited significantly less time spent in the open sections and entries into the open sections, respectively, compared to the control group during Test 1 (one-way ANOVA: time spent, *F*_(4, 92)_ = 12.69, *p* < 0.05; number of entries, *F*_(4, 92)_ = 12.288, *p* < 0.05; Fig. 1d, e). These observations confirmed previous findings and showed that SD mice exhibited anxiety-like behavior [8–10].

We next examined the effects of MEM treatment. Two-way ANOVA revealed significant effects of drug (VEH vs. MEM: avoidance zone, *F*_(3, 57)_ = 12.188, *p* < 0.05; interaction zone, *F*_(3, 57)_ = 6.653, *p* < 0.05) and time (Test 1 vs. Test 2: avoidance zone, *F*_(3, 57)_ = 76.186, *p* < 0.05; interaction zone, *F*_(3, 57)_ = 66.835, *p* < 0.05), and a drug × time interaction (avoidance zone, *F*_(3, 57)_ = 5.272, *p* < 0.05; interaction zone, *F*_(3, 57)_ = 5.51, *p* < 0.05; Fig. 1b, c). Importantly, post hoc Bonferroni’s analysis revealed that MEM-treated SD mice exhibited significantly less and more time spent in the avoidance and interaction zones, respectively, than VEH-treated SD mice during Test 2 (*ps* < 0.05; Fig. 1b, c), indicating that MEM-treated SD mice exhibited less social avoidance behavior. MEM-treated SD mice exhibited comparable time spent in the avoidance and interaction zones as the control groups (*ps* > 0.05; Fig. 1b, c), suggesting that these mice showed comparable social behavior with the control groups. Taken together, our observations suggest that MEM treatment induces the forgetting of social avoidance memory.

We next asked whether forgetting of social avoidance memory is associated with an improvement of anxiety-like behavior. Two-way ANOVA revealed significant effects of drug (VEH vs. MEM: time spent, *F*_(3, 57)_ = 12.808, *p* < 0.05; number of entries, *F*_(3, 57)_ = 19.569, *p* < 0.05) and time on the number of entries (Test 1 vs. Test 2: time spent, *F*_(3, 57)_ = 0.909, *p* > 0.05; number of entries, *F*_(3, 57)_ = 16.766, *p* < 0.05; Fig. 1b) and drug × time interactions (time spent, *F*_(3, 57)_ = 20.816, *p* < 0.05; number of entries, *F*_(3, 57)_ = 16.766, p < 0.05; Fig. 1b, c). MEM-treated SD mice showed more time spent in the open sections and entries into the open sections than VEH-treated SD mice during Test 2 (*ps* < 0.05); moreover, their scores were comparable with control mice (*ps* > 0.05; Fig. 1d, e). These observations indicate that MEM-treated SD mice showed normal anxiety-like behavior, suggesting that MEM treatment improves increased anxiety observed in SD mice.

**Fig. 2:**
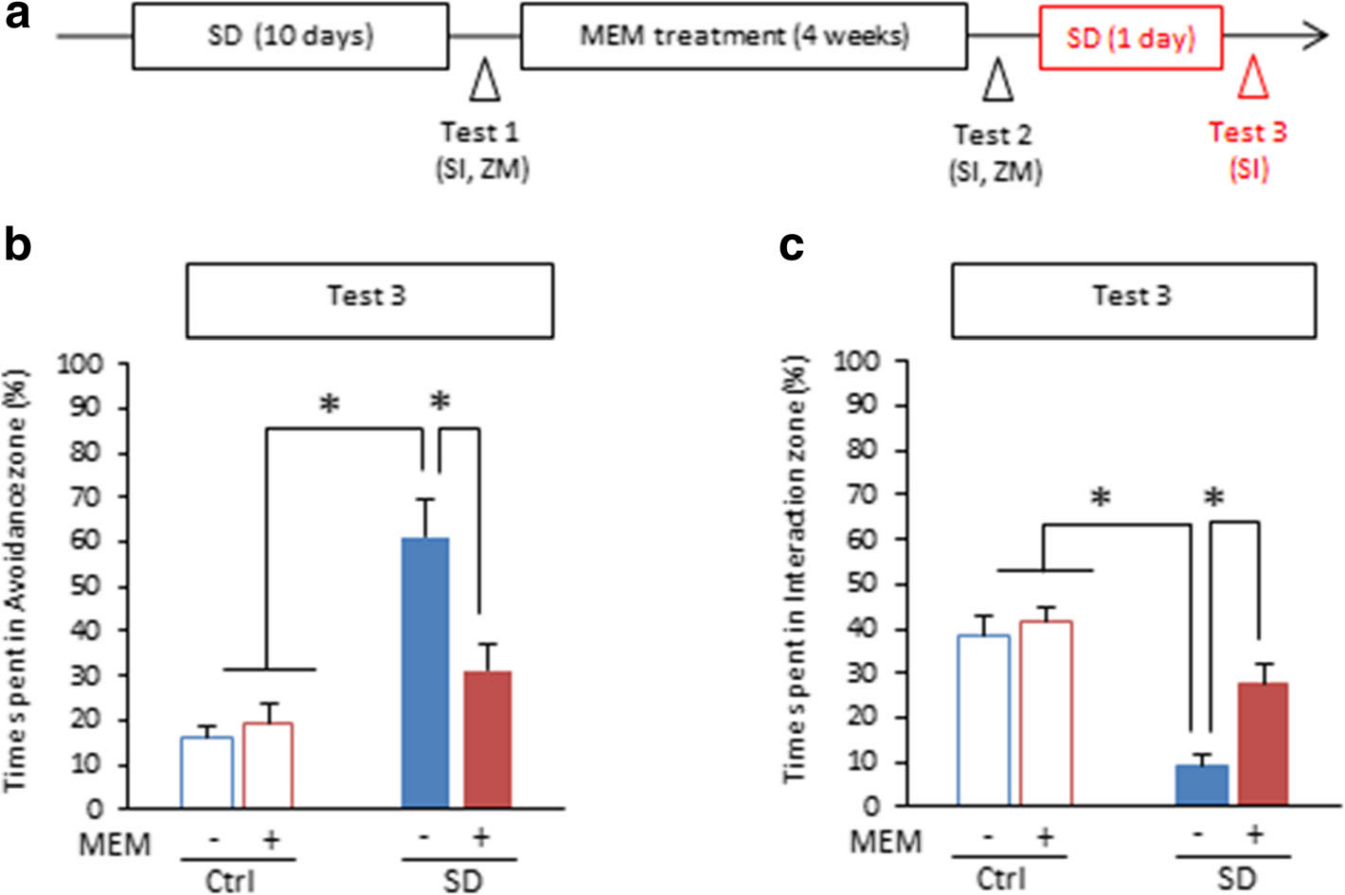
MEM-treated SD mice show no reinstatement of social avoidance memory following single re-exposure to aggressor. **a** Experimental procedure. **b** and **c** Social interaction task in Test 3. (VEH-Ctrl, *n* = 9; MEM-Ctrl, *n* = 8; VEH-SD, n = 8; MEM-SD, *n* = 6). Time spent in the avoidance zone (**b**) and interaction zone (**c**). *p < 0.05. Error bars indicate SEM

The possibility still remains that decreases in social avoidance behavior following MEM treatment is due to fear extinction learning since mice were exposed to the aggressor mice without SD treatment during social interaction task (Test 1 and Test 2). Therefore, we asked whether MEM treatment induces forgetting or allows the reinstatement of social avoidance memory after treatment since the reemergence of a previously extinguished fear (reinstatement) occurs in rodents and humans [11, 12]. We performed the social interaction task again after a single re-exposure to the aggressor mice following Test 2 (Test 3; Fig. 2a). Control mice were not exposed to the aggressor but tested as SD mice. Two-way ANOVA revealed significant effects of drug (VEH vs. MEM: avoidance zone, *F*_(3, 30)_ = 5.216, *p* < 0.05; interaction zone, *F*_(3, 30)_ = 7.549, *p* < 0.05), SD (Ctrl vs. SD: avoidance zone, *F*_(3, 30)_ = 23.734, *p* < 0.05; interaction zone, *F*_(3, 30)_ = 31.147, *p* < 0.05), and a drug × SD interaction for time spent in the avoidance zone (avoidance zone, *F*_(3, 30)_ = 7.776, *p* < 0.05; interaction zone,* F*_(3, 30)_ = 3.629, *p* = 0.068; Fig. 2b, c). Importantly, VEH-treated SD mice exhibited significantly more and less time spent in the avoidance and interaction zones, respectively, than the other groups (*ps* < 0.05; Fig. 2b, c). In contrast, MEM-treated SD mice still showed comparable social avoidance behavior with the control groups (time spent in avoidance and interaction zone, MEM-Ctrl vs. MEM-SD, *ps* > 0.05). Therefore, these observations suggested that MEM-treated SD mice did not show reinstatement of social avoidance memory and supported our conclusion that MEM treatment induced the forgetting of social avoidance memory. It is important to note that MEM-treated SD mice showed significant increase in time spent in avoidance zone at Test 3 compared to Test 2 (*p* < 0.05). However, our observation that even a single SD treatment (as a first SD trial during SD treatment for 10 days) significantly increased time spent in the avoidance zone (data not shown) suggests that single SD is enough to induce social avoidance behavior.

**Fig. 3:**
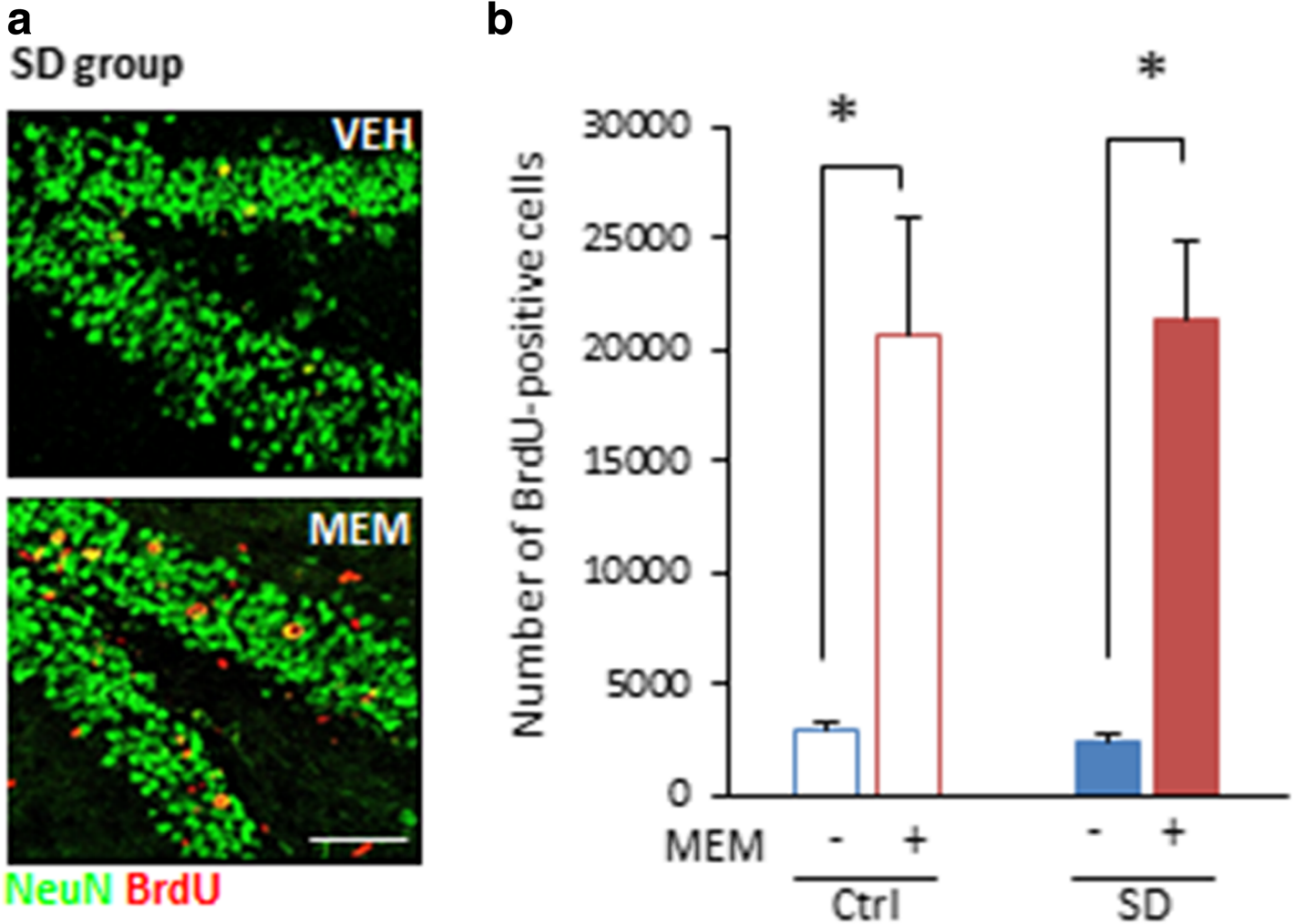
MEM treatment enhances adult hippocampal neurogenesis in SD mice. **a** Representative immunofluorescent staining of BrdU-positive cells (red) and NeuN-positive cells (green) in VEH-treated (top) or MEM-treated (bottom) SD mice. Scale bar indicates 100 μm. **b** Quantification of BrdU-positive cells (VEH-Ctrl, n = 3; MEM-Ctrl, n = 3; VEH-SD, n = 3; MEM-SD, n = 3). *p < 0.05. Error bars indicate SEM

MEM treatment promotes adult hippocampal neurogenesis [5, 6]. To examine the effects of MEM treatment on hippocampal neurogenesis in SD mice, mice received systemic injections of 5-bromo-2-deoxyuridine (BrdU; 50 mg/kg body weight) to label proliferating cells, at 2 days after each MEM or VEH treatment and then the number of BrdU-positive cells was quantified at 24 h after Test 3 using immunohistochemistry (Fig. 3a). Two-way ANOVA revealed a significant effect of drug (drug, *F*_(3, 15)_ = 68.159, *p* < 0.05; SD, *F*_(3, 15)_ = 0.001, *p* > 0.05; drug × SD interaction, *F*_(3, 15)_ = 0.073, *p* > 0.05; Fig. 3b). Both MEM-treated groups exhibited significantly more BrdU-positive cells than the VEH-treated groups (*ps* < 0.05; Fig. 3b). Thus, MEM treatment enhanced adult hippocampal neurogenesis in SD mice.

In this study, we used an SD stress paradigm as a PTSD-like model and examined the effects of neurogenesis enhancer MEM on SD stress-induced PTSD-like behavior including social avoidance memory and anxiety-like behavior. We found that MEM treatment enhanced the forgetting of social avoidance memory. Importantly, reinstatement of social avoidance memory was not observed following single re-exposure to the aggressor mouse (Fig. 2), suggesting that MEM treatment enhanced forgetting of SD memory rather than SD memory extinction. Interestingly, previous study showed that the optogenetic activation of ventral hippocampus increased social avoidance behavior [13]. Therefore, it is possible that forgetting of SD memories stored in the ventral hippocampus were facilitated by the increase in ventral hippocampal neurogenesis. More importantly, MEM treatment improved anxiety-like behavior. These observations suggest that the increase of adult hippocampal neurogenesis by MEM improves anxiety-related behavior through the forgetting of social avoidance memory. Our findings raise the possibility that neurogenesis enhancers improve PTSD-like behavior by facilitating the forgetting of traumatic memory. However, it is important to note that the possibility was not excluded that enhancement of hippocampal neurogenesis improved anxiety directly, but not indirectly through forgetting [14, 15]. Further investigations are required to examine the relationship between forgetting of fear memory and improved anxiety-like behavior.

## Methods

### Animals

All experiments were conducted according to the *Guide for the Care and Use of Laboratory Animals of the Japan Neuroscience Society* and the *Guide of the Tokyo University of Agriculture*. All animal experiments were approved by the Animal Care and Use Committee of Tokyo University of Agriculture. Male C57BL/6 N mice were obtained from Charles River (Yokohama, Japan). The mice were housed in cages of 6, maintained on a 12-h light/dark cycle, and allowed ad libitum access to pellet food and water. The mice were at least 6 weeks of age at the start of the experiments, and all behavioral procedures were conducted during the light phase of the cycle. ICR male mice that had been retired from breeding were purchased from Charles River as aggressor mice. All experiments were conducted blind to the treatment condition. Animal behavior was recorded using a video camera.

### Drug

MEM (Sigma, MO, USA) was dissolved in 0.9% saline. Mice were injected intraperitoneally with MEM at a dose of 50 mg/kg body weight once a week for 4 weeks. Control mice were injected with the same volume of 0.9% saline. At 2 days after each MEM injection, the mice received a single injection with 50 mg/kg body weight of BrdU (Sigma).

### SD stress

Repeated SD stress was performed as described previously [8–10]. Male C57BL/6 mice to be defeated were isolated for a week and exposed an aggressor ICR mouse for 10 min daily for 10 consecutive days. The pair of defeated and aggressor mice was randomized. Control mice (no SD mice) were exposed in an empty cage for 10 min daily for 10 consecutive days. To examine the effects of MEM treatment, the SD resilient mice were categorized and excluded as previous experiments [10]. Resilient mice were defined as those that showed time spent in interaction and avoidance zones for more than 60% and less than 40%, respectively [10]. ICR mice were screened based on their aggressiveness to a naïve C57BL/6 mouse, as measured by the latency and number of attacks, and the mice showing stable aggression were used as aggressor mice for repeated SD stress.

### Social interaction task

The social interaction task was performed as described previously [8–10]. An ICR mouse enclosed in a wire mesh cage was placed into the open field chamber (50 × 50 × 40 cm). The test mouse was allowed to explore the chamber freely for 5 min. The time spent in the interaction zone (area near the aggressor mouse) and avoidance zone (two corners opposite to the aggressor mouse) was measured using an automatic monitoring system (O’Hara & Co., Ltd., Tokyo, Japan).

### Elevated zero maze task

The zero maze consisted of a circular path (5.5 cm width, inner diameter of 46 cm) that had two open and two closed sections (walls were 15 cm high) and was elevated 50 cm above the floor. Mice were initially placed in the closed section and their behavior was measured for 5 min. The length of time spent in the open sections and the number of times they entered into the open sections with two or four paws were measured [16, 17].

### Immunohistochemistry

Immunohistochemistry was performed as described previously [6, 18]. After anesthetization, all mice were perfused with 4% paraformaldehyde containing 0.5% picric acid. The brains were removed, fixed overnight, transferred to 30% sucrose, and stored at 4 °C. Coronal sections (14 μm) were generated using a cryostat. Consecutive sections were boiled in citrate buffer solution for 5 min and incubated with 2 N HCl at 37 °C for 30 min, followed by incubation in a blocking solution. The sections were incubated overnight with a monoclonal rat anti-BrdU primary antibody (1:5000; Novus Biologicals, Littleton, CO) and a monoclonal mouse anti-NeuN primary antibody (1:500; Millipore, Hayward, CA) in the blocking solution. Subsequently, the sections were incubated for 2 h with Alexa Fluor 594-conjugated goat anti-rat IgG (1:500; Invitrogen, Grand Island, NY) and Alexa Fluor 488-conjugated goat anti-mouse IgG (1:500; Invitrogen).

### Quantification

All fluorescence images were acquired using a confocal microscope (TCS SP8; Leica, Wetzlar, Germany). Equal cutoff thresholds were applied to all slices using LAS X software (Leica). BrdU-positive cells throughout the rostro-caudal extent of the dentate gyrus (Bregma − 0.94~ − 4.04 mm; from dorsal to ventral) were counted in every eighth section, and the total number of BrdU-positive cells was calculated by multiplying the count in each section by 8 and then totaling the values. BrdU-positive cells were colocalized with NeuN, a marker of mature neurons, and the number of these cells was counted.

### Data analysis

One-way or two-way ANOVA followed by post hoc Bonferroni’s comparisons were used to analyze the effects of drug, time, and SD stress. All values in the text and figure legends represent the mean ± standard error of the mean (SEM).

## Data Availability

Please contact author for data requests.

## Abbreviations

BrdU: 5-bromo-2-deoxyuridine
MEM: Memantine
PTSD: Post-traumatic stress disorder
SD: Social defeat
SI: Social interaction task
ZM: Elevated zero maze task
